# Effect of Unilateral Congenital Ptosis on Ocular Higher Order Aberrations in Children

**Published:** 2013

**Authors:** Dhivya Ashok Kumar, Amar Agarwal MS, Gaurav Prakash, Neha Vajpayee Boptm, Sathiya Packiyalakshmi, Athiya Agarwal

**Affiliations:** Dr Agarwal’s Eye Hospital and Eye Research Centre, Chennai, India

**Keywords:** Congenital ptosis, Ocular higher order aberrations, Children

## Abstract

To analyse the effect of congenital unilateral ptosis on the ocular higher order aberrations (HOA) and to compare these eyes with normal fellow eyes this study has been performed. In this observational comparative case series, 16 eyes of 16 patients less than 15 years old with congenital unilateral upper eyelid ptosis were included. Corrected distance visual acuity (CDVA), corneal topography, ocular HOA’s with Zywave workstation was recorded. The amount of ptosis was measured from marginal reflex distance (MRD1). The ocular HOA’s were compared between the ptosis and the normal fellow eyes after making necessary corrections to avoid errors due to enantiomeric midline symmetry. The mean age was 12.5±2.7years (range7-15years). The mean MRD1 was -0.9±1.8mm in the ptosis eyes. There was significant difference noted in the mean 6mm Zernicke coefficients Z_3_^−3^ (p=0.002), Z_4_^−2^ (p=0.034), Z_4_^2^ (p=0.008), Z_5_^−5 ^(p=0.044), Z_5_^1 ^(p=0.039), Z_5_^3 ^(p=0.036), Z_5_^5^ (p=0.044) between the ptosis and the fellow eyes. The mean Z3−3 was -0.17±0.15 and 0.07±0.12 in the ptosis and the normal eyes respectively. There was a significant difference (p=0.023) in total RMS (root mean square) between the ptosis and the normal eyes. Total coma aberration correlated with CDVA (p=0.004) and MRD (p=0.030) in the ptosis eyes. There was no correlation (p=0.815) between the age (duration of ptosis) and total RMS. In conclusion, Eyes with congenital ptosis differed from their normal fellow eyes in the higher order aberrations. None of the HOA’s which differed between the two groups affected the visual acuity in the ptosis eyes.

## INTRODUCTION

Congenital upper eyelid ptosis is characterized by the drooping of upper eyelid since childhood due to poor development of the levator muscle. Corneal topographic changes and induced astigmatism have been reported with ptosis in former studies [1-2]. A higher incidence of amblyopia has been known with congenital ptosis [3-4]. Normal upper eyelid is known to induce static pressure on contact with the ocular surface [5]. Drooping upper eyelid pressure can cause changes in the ocular surface especially on the cornea. Till now, the effect of ptosis on ocular higher order aberrations and its impact on visual acuity have been unidentified. An inter-ocular difference in the higher order wavefront patterns between the amblyopic and normal eye has been shown to affect the visual acuity [6-8]. However, Kirwan et al reported that unlike lower order aberrations such as sphere and cylinder, higher order aberrations are unlikely to play a role in the development of amblyopia [9]. Though higher order aberrations analysis in children has been reported in various situations [9-12] there are few reports on the analysis on the effects of congenital ptosis on the ocular higher order aberrations (HOA) [13]. In this prospective study, we have analyzed the HOA profile in eyes with unilateral congenital ptosis and compared them with normal fellow eyes in children less than 15 years old.

## METHODS

Our study is an observational comparative prospective case series. Informed consent was taken from all patients’ guardians. The study has been approved by Institutional Review Board of our hospital, namely the Academics and Research Board. Pediatric patients (less than 15 years old) with unilateral congenital ptosis were recruited from oculoplastic specialty clinic of Dr. Agarwal’s Eye Hospital and Eye Research Centre from 2010 January through September 2011. Patients with complicated congenital ptosis, tear film abnormality, abnormal bells phenomenon, high ametropia more than 2D and those with a history of previous ocular surgery were excluded. We used Snellen’s distance vision acuity charts and visual acuity was presented in decimal equivalent.

**Ptosis evaluation**

The accurate position of the upper eyelid was determined by assessing the primary gaze in the repose position. The marginal reflex distance (MRD1) defined as the distance from the central light reflex on the cornea to the upper eyelid margin in the primary position. Other ptosis work which includes Vertical fissure height (VFH), levator muscle function, corneal sensation, Bell’s phenomenon, extra ocular movements, slit lamp biomicroscopy, fundus examination and tear film abnormality tests (Schirmer’s test, Break up time and corneal staining) were performed in all eyes. Corneal topography was recorded with computed topographic system (Orbscan, Bausch & Lomb, Rochester, NY). Corrected distance visual acuity (CDVA) and uncorrected distance visual acuity in Snellen visual acuity charts was measured in both eyes. 

**Aberration profile examination**

Ocular higher order aberrations were measured using the Zywave workstation (Bausch & Lomb, Rochester, NY), a device based on the Hartmann- Shack principle. All aberrometry measurements were taken between 2-6 PM to compensate for diurnal variation. Aberrometry readings were taken in both (ptosis and fellow) eyes of the patients by a single examiner. Homatropine 2% eye drops were used uniformly for dilatation and mydriasis during the dilated aberrometry examination. For analysis purposes, higher order aberrations at 6mm pupil size was taken as standard for all eyes. Patients were asked to blink twice to smooth the corneal surface to give a reproducible measurement. For uniform measurements, the upper eyelid was lifted in all eyes mechanically just prior to aberrometry examination to visualize the centroids. The result table of the Zywave station automatically would select the best three readings with the lowest reliability criteria (the lower the reliability criteria, the better the repeatability). The numerical values of Zernike polynomials were taken from ‘.ate’ files and band plots. These were then converted to the standard nomenclature of the Optical Society of America and entered into a Microsoft excel sheet (Microsoft Corp, Redmond, Washington, US). Necessary corrections in the signed values were made according to recommendations of Smolek, et al to avoid errors due to enantiomeric midline symmetry [14]. The sign of odd symmetric terms about the y axis was flipped in the left eye to combine data in a single database and also to test for mirror symmetry. The aberrations profile of ptosis eyes were then compared with the normal fellow eyes of the patients.

**Methods of literature search**

We have performed a Medline search with Pubmed. Ocular higher order aberrations (HOA) in ptosis, effect of congenital ptosis on HOA, ocular aberrations in congenital ptosis were some of the search terms used. The search was restricted to publications related to humans, in English and other language publications with English abstracts. Articles, book chapters, online abstracts obtained from the reference lists of other articles were reviewed and included when considered appropriate.

**Statistical analysis**

Data entered in a Microsoft Excel Sheet (Microsoft Corp, Redmond, Washington, US), and analyzed using SPSS version 16.1 (SPSS Inc, Chicago, Illinois, USA). Wilcoxon signed rank test was used for comparison with fellow eyes and Pearson bivariate correlation was used for correlation testing. Differences were considered statistically significant at p<0.05.

**Figure 1:**
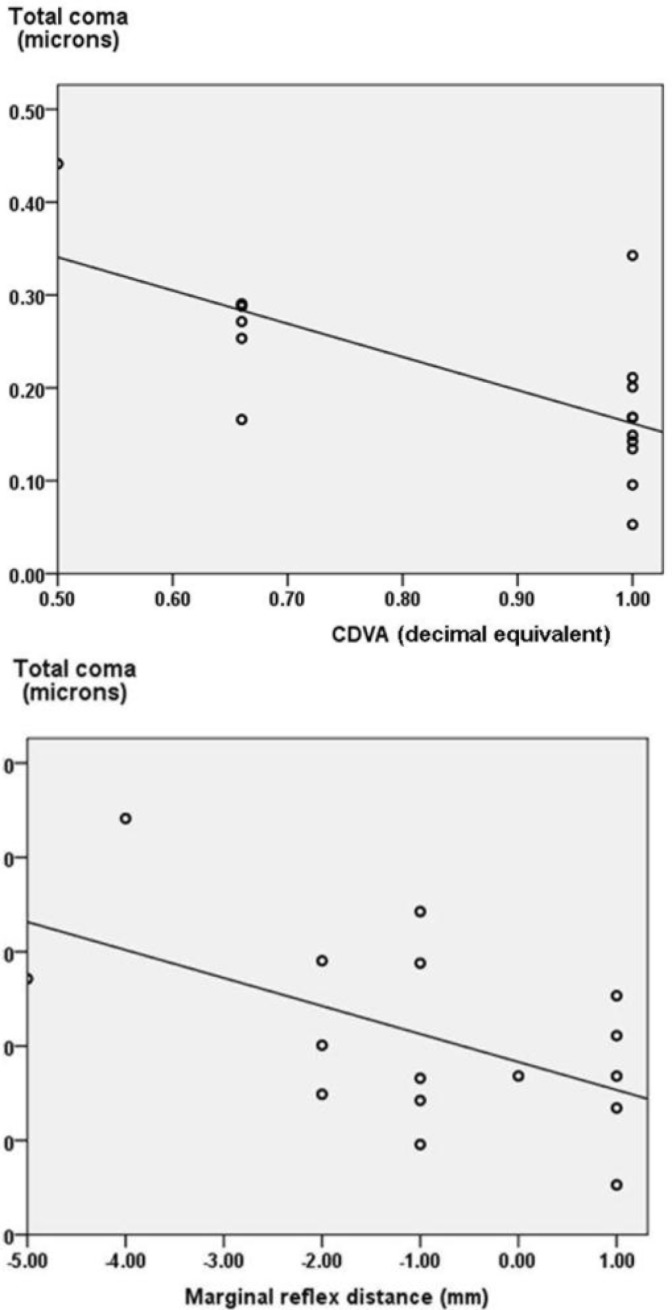
Scatter plot showing the correlation of total coma with corrected distant visual acuity (CDVA) in decimal equivalent and Marginal Reflex distance (MRD in mm) in ptosis eyes

## RESULTS

There were 16 eyes (4 right eyes and 12 left eyes) with congenital unilateral ptosis. The mean age was 12.5±2.7years (range 7-15 years old). The mean MRD1 was -0.9±1.8mm in the ptosis eyes. The mean VFH was 6.2±2.3 mm in the primary gaze. There was a significant difference between the ptosis and normal eyes in MRD (p=0.00) and VFH (p=0.00). The mean spherical equivalent was -0.76±1.4 in the congenital ptosis. There was no significant difference (p=0.850) in the mean spherical equivalent between the ptosis and the fellow eyes. The mean CDVA was 0.86±0.18 in ptosis eyes. There was significant difference in CDVA (p=0.034) between the ptosis and their normal fellow eyes. There was significant correlation between CDVA and MRD (p=0.037, r= 0.524) in the ptosis eyes.The mean and standard deviations of higher order aberrations from third to fifth order aberrations Zernicke terms were shown in Table 1. There was a significant difference (p=0.023) in total RMS (root mean square) between the ptosis and the normal eyes (Table 2). However, there was no difference noted in 6mm higher order RMS (p=0.959). Seven out of 15 Zernicke’s term from 3^rd^ to 5^th^ differed between the ptosis and the fellow eyes (Table 1). There was significant difference (p=0.002) noted in the third order Zernicke coefficient vertical trefoil Z_3_^−3 ^between the ptosis and the fellow eyes (Table 1). The mean Z_3_^−3 ^was -0.17±0.15 and 0.07±0.12 in the ptosis and the normal eyes respectively. There was no significant difference in vertical or horizontal coma. There was no difference noted in Total Coma (Z_3_^1^, Z_3_^-1^, Z_5_^-1^, Z_5_^1^), and Total Trefoil (Z_3_^-3^, Z_3_^3^, Z_5_^-3^, Z_5_^3^) between the eyes. There was significant difference in the fourth order Z_4_^−2^ (p=0.041) and Z_4_^2^ (p=0.027) coefficients as well. There was no significant difference (p=0.098) in the spherical aberration between the ptosis and the normal fellow eyes. The mean values comparison of various other fourth order aberrations are given in (Table 1). A significant correlation was noted with total coma with MRD and CDVA (Figure 1, Table 3) in the ptosis eyes. None of the Zernicke terms which differed between the ptosis and the fellow eyes showed correlation with CDVA or MRD (Table 3). There was no correlation (p=0.815) between the age (duration of ptosis) and total RMS. 

**Table1:** Difference in the individual Zernicke terms between the congenital ptosis eyes and their normal fellow eyes

**ZERNICKE term**	**Ptotic eye**		**Fellow eye**		
	Mean	SD	Mean	SD	P value
Z_3_^−3^	-0.17	0.15	0.07	0.12	**0.002**
Z_3_^−1^	-0.04	0.20	-0.04	0.22	0.535
Z_3_^1^	-0.08	0.09	-0.07	0.12	0.717
Z_3_^3^	-0.14	0.26	-0.01	0.13	0.070
Z_4_^−4^	0.04	0.07	-0.005	0.05	0.121
Z_4_^−2^	0.03	0.05	-0.0004	0.04	**0.034**
Z_4_^0^	0.05	0.11	-0.03	0.12	0.098
Z_4_^2^	0.05	0.06	0.01	0.05	**0.008**
Z_4_^4^	0.03	0.05	0.003	0.03	0.148
Z_5_^−5^	-0.04	0.05	-0.004	0.02	**0.044**
Z_5_^−3^	-0.02	0.04	0.007	0.03	0.088
Z_5_^−1^	-0.03	0.04	-0.01	0.03	0.156
Z_5_^1^	-0.02	0.02	0.002	0.03	**0.039**
Z_5_^3^	-0.02	0.01	-0.007	0.02	**0.036**
Z_5_^5^	-0.04	0.05	-0.004	0.02	**0.044**

Z: Zernicke coefficients, SD: Standard Deviation

**Table 2:** Comparison of higher order aberrations between ptosis and fellow eyes

	Ptosis eye	Fellow eye	P value
Total RMS 3RD order RMS 4^th ^order RMS 5^th^ order RMS Total TREFOIL Total COMA Spherical aberration	2.02±1.70 0.35±0.25 0.17±.080 0.08±.060 0.26±0.26 0.21±0.10 0.05±0.10	1.33±1.10 0.28±0.16 0.13±0.07 0.05±0.03 0.16±0.10 0.21±0.10 -0.02±0.10	**0.023** 0.326 0.148 0.088 0.352 1.000 0.098

RMS: Root mean square

**Table. 3:** Correlation of higher order aberrations with visual acuity and ptosis severity

	Correlation Coefficient	P value
Total coma-CDVA Total coma-MRD Total trefoil-CDVA Total trefoil-MRD Vertical trefoil -CDVA Vertical trefoil -MRD Z_4_^−2^-CDVA Z_4_^-2^-MRD Z_4_^2^-CDVA Z_4_^2^-MRD Z_5_^-5^-CDVA Z_5_^-5^-MRD Z_5_^1^-CDVA Z_5_^1^-MRD Z_5_^3^-CDVA Z_5_^3^-MRD	-0.677 -0.542 0.059 0.039 0.330 0.280 0.200 0.074 0.074 -0.492 0.034 0.384 -0.395 -0.187 -0.132 0.039	**0.004** **0.030** 0.827 0.887 0.216 0.300 0.461 0.787 0.787 0.053 0.900 0.142 0.130 0.488 0.626 0.886

CDVA: Corrected distant visual acuity (decimal equivalent), MRD: Marginal reflex distance (mm)

## DISCUSSION

With the recent developments in technology and investigative modalities there has been a large interest in higher-order aberrations in various eye conditions. Higher order aberrations in children have been studied widely in the recent past [8-12]. Kirwan et al in his study on HOA’s in children showed increased ocular aberrations in myopic eyes [10]. They postulated that the higher level of ocular aberrations was a stimulus for inducing myopia [11]. Qui et al in his study reported that lower order aberrations are the main refractive factors leading to amblyopia [12]. However they noticed that HOA’s increase with the increasing degree of anisometropic amblyopia. Kirwan and his associates noted that the mean root mean square values of higher order aberrations did not significantly differ between normal and amblyopia eyes [9]. On further literature search, we noticed that Sussenbach et al reported that upper eyelid ptosis masked the existing irregular astigmatism including vertical coma and the ptosis surgery unveiled the changes leading to alteration in the optical wavefront and resultant image degradation [13]. To our knowledge there are no case series on the evaluation of changes of higher order aberration in congenital ptosis. 

Brunette and associates conducted a study that investigated the effect of age on higher-order aberrations from five years to old age and concluded that the development of the optical structures of the eye were associated with a reduction in higher-order aberrations [15]. It has been known that the change in eyelid position during daily activities can affect corneal topography and ocular aberrations [16-19]. Collin et al proposed that the eyelid-induced corneal aberrations may play a role in myopia development [16]. Eyelid position during the tasks is an important factor which makes the difference in the aberrations. Beuhren et al reported that the changes observed in corneal topography after reading was directly related to the force exerted by the eyelids during reading [17]. According to Han et al there was a difference in the Zernicke coefficient of the defocus, 90/180 astigmatism, vertical coma and spherical aberration between the narrowed and wide-open lid positions [18]. A report by Bheuren et al showed that myopes had greater levels of some high order ocular wavefronts than the emmetropes and attributed it to the narrowed palpebral lid aperture while reading [19].

In our series, there was definite decrease in palpebral aperture height in the ptosis eyes. It was noted that Z_3_^−3 ^ in the third order, Z_4_^−4^, Z_4_^2^ in the fourth order and Z_5_^−5^, Z_5_^1^, Z_5_^3^, Z_5_^5^ in the fifth order differed significantly between the ptosis and normal fellow eyes. Though coma did not differ significantly between the 2 groups, there was correlation of total coma with CDVA and MRD (Figure 1). Surprisingly, the vertical trefoil (Z_3_^−3^) which differed between the 2 groups did not show significant correlation with CDVA or MRD. As we know that vertical orientation of trefoil is significantly more frequent than the opposite in normal eyes and it has been shown that the interaction of vertical trefoil with coma improved the visual acuity more than when they acted independently. [20]. Lack of mirror-image symmetry with the fellow eye was known to be higher in eyes associated congenital ptosis and amblyopia [1]. As we know, the causes of amblyopia in ptosis can be astigmatism, anisometropia, strabismus and stimulus deprivation. In our study, the CDVA showed significant difference between the ptosis and normal eyes; only total coma showed significant correlation with CDVA in ptosis. However other aberrations which differed from the normal eyes (Table 3) do not show significant correlation. The CDVA of congenital ptosis eyes correlated with the MRD (p=0.037) measured. Thus, the eyes with severe ptosis with low MRD had poor vision. This finding showed that stimulus deprivation was the major cause of less vision in the ptosis eyes. Though uncompensated, higher order aberrations can cause subnormal vision [21]. In our study the higher order aberrations did not significantly affect the vision in ptosis eyes. 

Though there is narrowed palpebral aperture in congenital upper eyelid ptosis, unlike myopic cases these patients do not squeeze their eyes. There is uniform pressure distribution in congenital ptosis unlike mechanical drooping of upper eyelid where there is localized pressure on the eyeball. Moreover, most of these unilateral congenital patients develop head positions which again will prevent excess upper eyelid pressure. These may be the reasons for the absence of significant difference in the third order HOA’s like Z_3_^−1^Z_3_^1 ^Z_3_^3 ^of the ptosis and the normal fellow eyes. Paralysis of accommodation appeared to have little effect on higher-order aberrations in children [10]. Hence dilatation at 6mm pupil has been taken as standard for analysis. Tear film is an important ocular factor which is known to change in ptosis eyes [22-23]. Good Bell’s phenomenon is required to maintain normal tear film stability [23]. Thus patients with abnormal Bells with ptosis can have tear film abnormality as well as changes in ocular aberrations. However, in our study, all eyes had good Bells phenomenon and there was no tear film abnormality. Small sample size can affect the power of the analysis, which is the limitation of this study. Moreover visual acuity examination following compensation of HOA’s was not attempted.

## CONCLUSION

There were few coefficients (Z_3_^−3^, Z_4_^−2^, Z_4_^2^, Z_5_^−5^, Z_5_^1^, Z_5_^3^, Z_5_^5^) which differed between the normal fellow eyes and the ptosis eyes. Out of all the higher order aberrations’s (HOA), only total coma correlated with CDVA and MRD of the ptosis eyes. We recommend further study on the detailed analysis HOA’S in ptosis eyes after surgical correction. A further evaluation in a large population and serial follow up comparison after surgical correction is essential. Nevertheless this study will let us think on a new prospect of ocular aberration variations in eyes with congenital ptosis, a common eyelid problem in oculoplastics.

## DISCLOSURE

Prof Amar Agarwal is a paid consultant for Staar surgicals. Rest of the coauthors: None declared.
